# Markedly Improved Glycemic Control in Poorly Controlled Type 2 Diabetes following Direct Acting Antiviral Treatment of Genotype 1 Hepatitis C

**DOI:** 10.1155/2016/7807921

**Published:** 2016-05-17

**Authors:** Raymond Anthony Pashun, Nicole T. Shen, Arun Jesudian

**Affiliations:** ^1^Department of Medicine, NewYork-Presbyterian Hospital, 505 East 70th Street, Suite 450, New York, NY 10021, USA; ^2^Division of Gastroenterology and Hepatology, NewYork-Presbyterian Hospital, Weill Cornell Medical College, 1305 York Avenue, 4th Floor, New York, NY 10021, USA

## Abstract

Type 2 diabetes mellitus (T2DM) is often associated with hepatitis C virus (HCV) infection. Successful HCV treatment may improve glycemic control and potentially induce remission of T2DM. We report a case of an obese 52-year-old woman with mixed genotype 1a/1b HCV infection with compensated cirrhosis and a 10-year history of poorly controlled T2DM on insulin therapy. Following successful therapy with sofosbuvir, simeprevir, and ribavirin, her insulin requirements decreased and her glycosylated hemoglobin (HgA1c) normalized despite weight gain. This case suggests an association between HCV and T2DM and the potential for significant improvement in glycemic control with eradication of HCV.

## 1. Introduction

Type 2 diabetes mellitus (T2DM) is a common comorbid condition in approximately one-third of individuals with chronic hepatitis C virus (HCV) infection [1]. Recent data suggest that HCV infection directly impairs glucose metabolism and contributes to insulin resistance [2]. Treatment of genotype 1 HCV infection with interferon based regimens or direct acting antiviral agents (DAAs) has correlated with improved insulin sensitivity and glycemic control in both insulin dependent and noninsulin dependent T2DM [3–7]. We report a case of mixed genotype 1a/1b HCV infection with compensated cirrhosis treated with DAAs resulting in significant improvement of poorly controlled T2DM despite increasing body mass index (BMI).

## 2. Case

A 52-year-old morbidly obese woman with mixed genotype 1a/1b HCV infection secondary to intravenous drug use complicated by compensated cirrhosis developed T2DM with worsening control over the three years preceding HCV treatment. She was initially diagnosed with HCV infection in 2002 and developed T2DM in 2005. Despite a stable BMI of 38 kg/m^2^ and compliance with her antidiabetic regimen, her glycemic control became poor in 2011, with a glycosylated hemoglobin (HgA1c) of 10.9%. At this time, she was also found to have cirrhosis. The patient's T2DM medical regimen was uptitrated to insulin glargine 40 units daily and insulin aspart 8 units prior to meals. She instituted lifestyle modifications including an aerobic exercise regimen. Prior to starting HCV treatment in April 2014, her BMI had improved to 33.91 kg/m^2^. Despite this, her glycemic control worsened with an increase of HgA1c to 11.9%. She was treated with 12 weeks of sofosbuvir, simeprevir, and ribavirin and achieved 24-week sustained virologic response (SVR). Eight weeks after beginning treatment, her HgA1c dramatically improved to 6.1% with a BMI of 31.97 kg/m^2^ without further significant lifestyle modifications. Her insulin regimen was reduced to glargine 15 units and aspart 6 units with continued improvement in HgA1c. On most recent follow-up, 18 months after first undetectable level of HCV and 15 months after the end of treatment, her HgA1c continued to improve to 5.5%, despite an increase in BMI to 36.12 kg/m^2^.

## 3. Discussion and Conclusion

The relationship between HCV infection and T2DM remains controversial. The prevalence of diabetes mellitus did not differ in patients with and without HCV infection in the United States population in one study [8]. On the other hand, many studies support an association between HCV infection and T2DM. Patients with chronic HCV infection are more prone to develop T2DM when compared to both healthy controls and patients with other liver diseases [1]. The etiology of T2DM in HCV infected individuals has been postulated to result from either hepatogenous T2DM resulting from advanced liver disease or “classical” T2DM due to virally mediated insulin resistance [9]. This distinction bears significant clinical implications in which hepatogenous T2DM is less frequently associated with microangiopathy than “classical” T2DM.

Insulin resistance is presumed to underlie “classical” HCV-mediated T2DM, but the exact mechanism remains unknown. Studies show that HCV impairs glucose metabolism directly via viral proteins and indirectly by altering proinflammatory cytokine levels [2]. The HCV core protein prevents the insulin receptor substrate-1 (IRS-1) association with its insulin receptor by increasing IRS-1 degradation through upregulation of serine/threonine phosphorylation or increased activity of suppressor of cytokine signaling 3 (SOCS3) [10–12]. These direct actions on the insulin signaling pathway impair downstream signaling and appropriate regulation of glucose and its metabolism. Indirectly, HCV-mediated production of IL-6 and TNF-alpha proinflammatory cytokines from sinusoidal liver cells interferes with insulin signaling pathways, increasing gluconeogenesis [13].

Successful eradication of HCV infection can improve glucose metabolism and reduce insulin requirements. A prior case report noted complete remission of noninsulin dependent T2DM when genotype 1 HCV was successfully treated with pegylated-interferon alpha and ribavirin [3]. Another case report found improved glycemic control limited to the treatment phase in an insulin dependent T2DM patient with HCV who did not respond to therapy with interferon and ribavirin [4]. The association between HCV genotype and insulin resistance has also been investigated with one study revealing SVR induced reduction in insulin resistance in patients with genotype 1 HCV [5]. The evidence behind the effect of DAA therapy on T2DM is conflicting. The results of one study suggested that danoprevir's antiviral effects may restore insulin sensitivity in patients with genotype 1 HCV [6]. A second study found that HCV suppression with DAA therapy produced a marked improvement in glycemic control regardless of genotype [7]. Although these data suggest benefits of DAA on glycemic control, a contrasting study found that DAA therapy with sofosbuvir and ledipasvir leads to the development of new-onset T2DM [14].

In our case, significant improvement in diabetic control was observed after successful HCV treatment with DAA therapy. This improvement in insulin requirements and HgA1c persisted following viral clearance despite an increase in the patient's BMI. Control of T2DM is usually advised* prior* to HCV therapy in order to increase response rates [15]. However, with the advent of potent DAA therapies, viral eradication may be possible despite poor glycemic control and may actually be an effective means of improving diabetic control.

In summary, we present a case of poorly controlled insulin dependent diabetes where DAA therapy for mixed genotype 1a/1b HCV led to a dramatic improvement in glycemic control. The complex interplay between HCV infection and T2DM is important for the clinician to consider when determining appropriate treatment. Increased awareness of this association may ultimately result in improved outcomes for patients afflicted by both conditions. This case highlights one of the many benefits of HCV therapy with DAAs beyond prevention of liver-related complications.

## References

[1] Hammerstad S. S., Grock S. F., Lee H. J., Hasham A., Sundaram N., Tomer Y. (2015). Diabetes and hepatitis C: a two-way association. *Frontiers in Endocrinology*.

[2] Negro F., Alaei M. (2009). Hepatitis C virus and type 2 diabetes. *World Journal of Gastroenterology*.

[3] Doyle M.-A., Cooper C. (2015). Successful hepatitis C antiviral therapy induces remission of type 2 diabetes: a case report. *American Journal of Case Reports*.

[4] Tahrani A., Bowler L., Singh P., Coates P. (2006). Resolution of diabetes in type 2 diabetic patient treated with IFN-*α* and ribavirin for hepatitis C. *European Journal of Gastroenterology and Hepatology*.

[5] Thompson A. J., Patel K., Chuang W.-L. (2012). Viral clearance is associated with improved insulin resistance in genotype 1 chronic hepatitis C but not genotype 2/3. *Gut*.

[6] Moucari R., Forestier N., Larrey D. (2010). Danoprevir, an HCV NS3/4A protease inhibitor, improves insulin sensitivity in patients with genotype 1 chronic hepatitis C. *Gut*.

[7] Pavone P., Tieghi T., d'Ettorre G. (2016). Rapid decline of fasting glucose in HCV diabetic patients treated with direct acting antiviral agents. *Clinical Microbiology and Infection*.

[8] Ruhl C. E., Menke A., Cowie C. C., Everhart J. E. (2014). Relationship of hepatitis C virus infection with diabetes in the U.S. population. *Hepatology*.

[9] Vanni E., Bugianesi E., Saracco G. (2016). Treatment of type 2 diabetes mellitus by viral eradication in chronic hepatitis C: myth or reality?. *Digestive and Liver Disease*.

[10] Bose S. K., Ray R. (2014). Hepatitis C virus infection and insulin resistance. *World Journal of Diabetes*.

[11] Kawaguchi T., Yoshida T., Harada M. (2004). Hepatitis C virus down-regulates insulin receptor substrates 1 and 2 through up-regulation of suppressor of cytokine signaling 3. *The American Journal of Pathology*.

[12] Walsh M. J., Jonsson J. R., Richardson M. M. (2006). Non-response to antiviral therapy is associated with obesity and increased hepatic expression of suppressor of cytokine signaling 3 (SOCS-3) in patients with chronic hepatitis C, viral genotype 1. *Gut*.

[13] Antonelli A., Ferrari S. M., Giuggioli D. (2014). Hepatitis C virus infection and type 1 and type 2 diabetes mellitus. *World Journal of Diabetes*.

[14] Premji R., Roopnarinesingh N., Qazi N., Nylen E. S. (2015). New-onset diabetes mellitus with exposure to ledipasvir and sofosbuvir. *Journal of Investigative Medicine High Impact Case Reports*.

[15] Ghany M. G., Strader D. B., Thomas D. L., Seeff L. B. (2009). Diagnosis, management, and treatment of hepatitis C: an update. *Hepatology*.

